# Behavior and psychological symptoms in dementia: could be predictors of biology?

**DOI:** 10.1192/j.eurpsy.2025.1663

**Published:** 2025-08-26

**Authors:** D. Castro, S. Orrego, M. Sava, M. Delso, M. P. García, A. M. Hualde, C. Terrón, M. D. S. Manzano Palomo

**Affiliations:** 1 Psychiatry, Infanta Leonor University Hospital; 2 Nuclear Medicine, Getafe University Hospital; 3 Nuclear Medicine, Gregorio Marañón University Hospital; 4 Neurology, Nuestra Señora del Rosario Hospital; 5 Neurology, Infanta Leonor University Hospital, Madrid, Spain

## Abstract

**Introduction:**

Neuropsychiatric symptoms (NPS), prevalent in individuals with mild cognitive impairment (MCI), are linked to functional decline, accelerated dementia progression, and reduced quality of life. In clinical practice, molecular imaging plays a key role in diagnosing cognitive and behavioral issues with high accuracy.

**Objectives:**

This study aims to analyze the correlation between NPS and molecular imaging findings in MCI-diagnosed patients.

**Methods:**

A retrospective, descriptive study was conducted with MCI patients who had undergone Amyloid PET scans (APscan) between January 2019 and October 2024 at Infanta Leonor Hospital in Madrid. Data included demographics, neurological diagnoses, Global Deterioration Scale (GDS) scores, NPS (e.g., depression, psychosis, behavioral and sleep disturbances, anxiety, suicidal thoughts), and PET-FDG/APscan results. Statistical analysis was performed using Dataset and SPSS 22.0.

**Results:**

A total of 72 patients were included. The main characteristics of the sample are shown in table 1. Among these patients, 65.28% exhibited NPS; notably, 49.3% had depression, 23.61% behavioral disturbances, 19.44% sleep disorders, 16.67% anxiety, 4.17% psychosis, and 2.82% suicidal ideation. In patients with a positive APscan, 29.79% had NPS, including 34.29% with depression and 66.67% with psychosis. Patients with abnormal FDG-PET scans showed higher NPS prevalence (65.96%), particularly behavioral disturbances (64.71%), sleep disorders (57.14%), and depression (62.86%).

**Image:**

**Figure:**
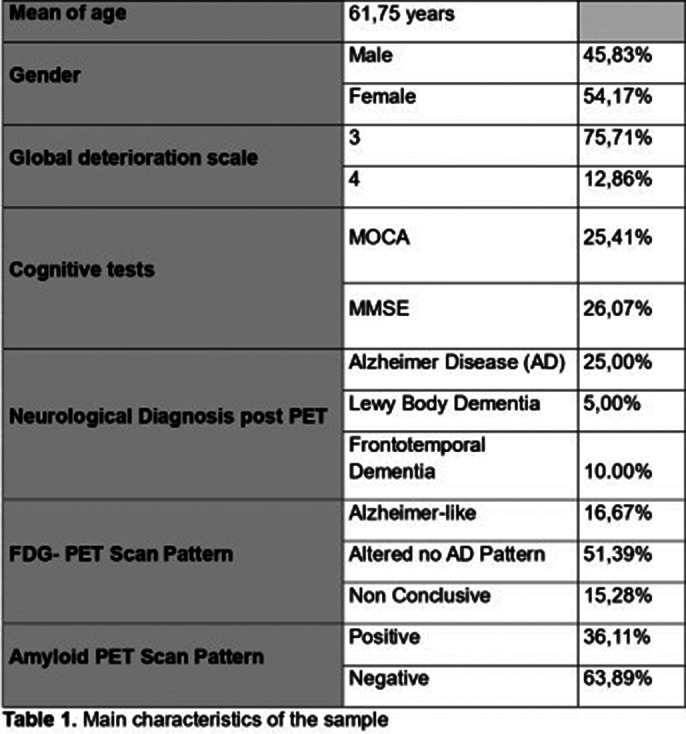

**Conclusions:**

This study underscores the high incidence of NPS in MCI patients, noting that NPS may exacerbate patient distress, contribute to autonomy loss, and increase institutionalization risk. Furthermore, molecular imaging patterns can help predict MCI progression to dementia and highlight NPS as potential predictors and outcomes of these biological changes.

**Disclosure of Interest:**

None Declared

